# A standardized flow cytometry network study for the assessment of circulating endothelial cell physiological ranges

**DOI:** 10.1038/s41598-018-24234-0

**Published:** 2018-04-11

**Authors:** Paola Lanuti, Pasquale Simeone, Gianluca Rotta, Camillo Almici, Giuseppe Avvisati, Rosa Azzaro, Giuseppina Bologna, Alfredo Budillon, Melania Di Cerbo, Elena Di Gennaro, Maria Luisa Di Martino, Annamaria Diodato, Paolo Doretto, Eva Ercolino, Alessandra Falda, Chiara Gregorj, Alessandra Leone, Francesca Losa, Natalia Malara, Mirella Marini, Pasquale Mastroroberto, Vincenzo Mollace, Michele Morelli, Emma Muggianu, Giuseppe Musolino, Arabella Neva, Laura Pierdomenico, Silvia Pinna, Giovanna Piovani, Maria Serena Roca, Domenico Russo, Lorenza Scotti, Maria Cristina Tirindelli, Valentina Trunzo, Roberta Venturella, Carlo Vitagliano, Fulvio Zullo, Marco Marchisio, Sebastiano Miscia

**Affiliations:** 1 Department of Medicine and Aging Sciences, University “G.d’Annunzio”, Chieti-Pescara, Italy; 2 Centre on Aging Sciences and Translational Medicine (Ce.S.I.-Me.T.), University “G.d’Annunzio”, Chieti-Pescara, Italy; 3 BD Biosciences Italia, 20090 Milano, Italy; 4 Laboratory for Stem Cells Manipulation and Cryopreservation, Department of Transfusion Medicine, Spedali Civili of Brescia, Brescia, Italy; 5 Hematology, Stem Cell Transplantation, Transfusion Medicine and Cellular Therapy, Department of Medicine, Campus Bio-Medico University Hospital, Roma, Italy; 6 Transfusion Service, Department of Hematology-Oncology and Stem Cell Transplantation Unit, Napoli, Italy; 7 Experimental Pharmacology Unit, Department of Research, Istituto Nazionale Tumori- IRCCS G. Pascale, Napoli, Italy; 8 Unit of Internal Medicine, Allergy and Clinical Immunology, Department of Medical Sciences “M. Aresu”, University of Cagliari Monserrato, Cagliari, Italy; 9 Clinical Pathology Laboratory, Department of Laboratory Medicine, AAS5, Pordenone Hospital, Pordenone, Italy; 10 Department of Health Science University “Magna Graecia” of Catanzaro, Catanzaro, Italy; 11 Laboratory for Stem Cells Manipulation and Cryopreservation, Department of Transfusion Medicine, Spedali Civili of Brescia, Brescia, Italy; 12 Department of Experimental and Clinical Medicine, University “Magna Graecia” of Catanzaro, Catanzaro, Italy; 13 Department of Obstetrics and Gynecology, University “Magna Graecia” of Catanzaro, Catanzaro, Italy; 14 Department Molecular Medicine and Translational, University of Brescia, Brescia, Italy; 15 Unit of Blood Diseases and Stem Cell Transplantation, University of Brescia, Brescia, Italy; 16 Department of Statistics and Quantitative Methods, University of Milano-Bicocca, Milano, Italy

## Abstract

Circulating endothelial cells (CEC) represent a restricted peripheral blood (PB) cell subpopulation with high potential diagnostic value in many endothelium-involving diseases. However, whereas the interest in CEC studies has grown, the standardization level of their detection has not. Here, we undertook the task to align CEC phenotypes and counts, by standardizing a novel flow cytometry approach, within a network of six laboratories. CEC were identified as alive/nucleated/CD45negative/CD34bright/CD146positive events and enumerated in 269 healthy PB samples. Standardization was demonstrated by the achievement of low inter-laboratory Coefficients of Variation (CV_L_), calculated on the basis of Median Fluorescence Intensity values of the most stable antigens that allowed CEC identification and count (CV_L_ of CD34bright on CEC ~ 30%; CV_L_ of CD45 on Lymphocytes ~ 20%). By aggregating data acquired from all sites, CEC numbers in the healthy population were captured (median_female_ = 9.31 CEC/mL; median_male_ = 11.55 CEC/mL). CEC count biological variability and method specificity were finally assessed. Results, obtained on a large population of donors, demonstrate that the established procedure might be adopted as standardized method for CEC analysis in clinical and in research settings, providing a CEC physiological baseline range, useful as starting point for their clinical monitoring in endothelial dysfunctions.

## Introduction

Circulating endothelial cells (CEC) represent a restricted peripheral blood (PB) cell subpopulation characterized by mature endothelial features. They detach from vessel walls, following vascular damage or the physiological tissue turnover, thus becoming circulating cells^1,2^. Interestingly, CEC have been proposed as a valuable biomarker in many diseases involving endothelium homeostasis (i.e. cardiovascular, inflammatory and metabolic pathologies, cancer, graft versus host disease onset in allogeneic hematopoietic stem cell transplantation) and as biomarker to monitor inhibition of angiogenesis in cancer treatment^3–5^. However, mostly due to their rareness and to their complex phenotype, different published techniques produced inconsistent results in terms of CEC quantification. Indeed, a broad range of CEC numbers (0–7900 cells/mL) has been detected by different authors in the PB of healthy donors^6^. Therefore, the development of a standardized approach for CEC evaluation and count results crucial in order to move their monitoring into the clinical practice. Of note, whereas the interest in CEC studies has grown exponentially in recent years, the standardization level of their identification and enumeration has not. In this context, polychromatic flow cytometry (PFC) is believed the most powerful technique for CEC evaluation. Recently, we have proposed a highly optimized PFC protocol for CEC identification and count^7^. By applying this protocol on a large population of healthy PB, a multi-site PFC study was here carried out by standardizing sample collection, reagents, protocols, instrument settings and data analysis. In order to guarantee the strict adherence to the established operating procedures, a through training of operators, and a real time data monitoring along the study were ensured, as already suggested^8^. The protocol has been applied, for the first time, to a large population of donors (N = 269); its robustness allowed the achievement of comparable results among centres, in terms of CEC identification and count. By aggregating data from multiple sites, CEC normality ranges and the relative biological variability were established.

## Materials and Methods

### Core network description and ethics committee that approved the study

A core network of six different Italian laboratories carried out the Standardization of Circulating Endothelial Cell evaluation (S.C.E.N.I.C. project). The involved laboratories are listed here below:

#### Site 1

Centre on Aging Sciences and Translational Medicine (CeSI-MeT), “University G.d’Annunzio” (CH, Italy); the study was approved by the ethic committee of the University “G.d’Annunzio”, Chieti-Pescara and of the “ASL N.2 Lanciano-Vasto-Chieti”, document “n.14 del 19.07.2012”;

#### Site 2

Interregional Research Center for Food Safety & Health (IRC-FSH), Department of Health Science, University “Magna Graecia” of Catanzaro (CZ, Italy); the study was approved by the ethic committee of the “Azienda Ospedaliera Universitaria Mater Domini, document N°2012.65 del 28.09.2012”;

#### Site 3

Clinical Pathology Laboratory, Department of Laboratory Medicine, “S. Maria degli Angeli” Hospital (PN, Italy); the study was approved by the ethic committee of the “ Azienda per i Servizi Sanitari n.6 Friuli Occidentale, document N°41121/DS”;

#### Site 4

Experimental Pharmacology Unit, Department of Experimental Oncology, National Cancer Institute - G. Pascale (Na, Italy); the study was approved by the ethic committee of the “Istituto Nazionale tumori Napoli, document N°699 del 02.08.2012”;

#### Site 5

Department of Transfusion Medicine, Laboratory for Stem Cells Manipulation and Cryopreservation, ASST Spedali Civili (BS, Italy); the study was approved by the ethic committee of the “Spedali Civili Brescia Azienda Ospedaliera Id NP 1195,document n° 39354 del 03.09.2012”;

#### Site 6

Department of Hematology, Stem Cell Transplantation, Transfusion Medicine and Cellular Therapy, Campus Bio-Medico University Hospital (RM, Italy); the study was approved by the ethic committee of the Policlinico Universitario, Campus Bio Medico di Roma, document n°66/12 27/11/2012.

All procedures were carried out under highly standardized conditions of protocols, reagents (same material/reagent lots) and flow cytometer instrument settings. The technical staff from all sites was trained and supported along the study (Supplemental Table 1). All methods were performed in accordance with the relevant guidelines and regulations.

### Donors

269 Caucasian healthy volunteers (age≥ 18 and ≤64 years) were enrolled from the sites of the core network (Supplemental Table 2), and 53 of them (N = 23 males and N = 30 females) were re-evaluated after 3 months.

In addition, 14 patients affected by malignant hematologic disorders (age ≥ 28 and ≤ 68 years) in complete remission (4 acute lymphocytic leukaemia, 8 acute myeloid leukaemia, 2 high-risk myelodysplastic syndrome) were enrolled by Site 5 and prospectively evaluated. All hematologic patients underwent allogeneic hematopoietic stem cell transplantation (allo-HSCT) from either HLA-matched familial (n = 6) or unrelated donors (n = 8); they received a standard myeloablative treatment (2 total body irradiation/Cyclophosphamide, 5 Busulphan/Cyclophosphamide) or a reduced intensity conditioning regimen (5 Busulphan/Fludarabine, 2 Busulphan/Fludarabine/Thiotepa). All patients and donors tested negative for HIV, HBV and HCV. Graft versus host disease prophylaxis was based on Cyclosporine/Methotrexate, plus AntiThymocyte Globulin treatments in patients receiving matched unrelated transplants. The assessment of CEC values was performed before (T1, pre-conditioning) and at the end (T2, pre-transplant) of the conditioning regimen.

Finally, 20 cutaneous systemic sclerosis patients (age ≥ 28 and ≤70 years) were recruited and evaluated by the “Unità Complessa Medicina Interna Allergologia e immunologia clinica, Azienda Ospedaliera Universitaria”, CA, Italy (Site 7). All these patients were females, since such a pathology is much more frequent in women than in men^9^; they were stratified according to the classification proposed by LeRoy^10^: 10 of the enrolled subjects resulted affected by limited cutaneous systemic sclerosis (lSSc) and 10 by diffuse cutaneous systemic sclerosis (dSSc). A further group of local healthy control subjects was recruited from Site 7 (n = 10; age ≥ 25 and ≤69 years, all females).

The study was approved by the local ethic committees. In accordance with the Helsinki II Declaration, all involved subjects gave written informed consent prior to their inclusion in the study, and participants were identified by specific codes. All patients were treated following the current clinical practice.

### Blood specimen collection

PB was drawn (21 G needles) in EDTA (Ethylenediaminetetraacetic acid) Vacutainer tubes (BD Biosciences, San Jose, CA, USA, cat. 368861). The first harvested 3 mL tube was excluded from the analysis, to avoid the effects of the vascular damage caused by venepuncture. Each first drawn PB tube was used to determine sample leukocyte count, to assess double platform counting.

In order to analyse the biological variability of CEC counts, 21 healthy subjects (10 females and 11 males, age ≥ 20 and ≤64 years) were enrolled by the core network sites for their multi-centre analysis; also in this case, each laboratory of the core network has enrolled a sub-group of healthy donors and PB samples were collected and analysed at four different time points (once a week, for four consecutive weeks). The analytical variability assessment was carried out by consecutively running two different stained samples from the same healthy donor.

### Flow cytometry

#### Instrument Standardization

A common instrument setup was obtained on each flow cytometer, in order to maximize fluorescence resolution sensitivity. Standard Deviation of Electronic background Noise (SDEN) was assessed for all fluorescent parameters on every instrument by Cytometer Setup and Tracking (CS&T) Beads and CS&T System (BD Biosciences). A SDEN matrix was then obtained, on the basis of the identification of the highest value for each fluorescent parameter. This matrix was used as a reference for all instruments involved in the study, in order to set up the photomultiplier gains so that the SDEN^2^ (variance) affected the total SDEN^2^, measured for unstained lymphocytes, by 10–20%. In order to set up photomultiplier gains, unstained cells were run on a single flow cytometer; CS&T beads were then acquired in the same conditions, in order to generate instrument setting target values, which were implemented on the other flow cytometers. All positive signals were detected within the linear range of the instruments. Flow cytometer performance, stability, and data reproducibility were sustained and daily checked in real time by using the CS&T quality control Module (BD Biosciences) and further validated by the acquisition of Spherotech 8 peaks Rainbow Beads (Spherotec. Lake Forest, IL, USA) and CS&T bright beads, after 30 minutes of laser power stabilization^11^. Median fluorescence intensity (MedFI) of Rainbow and CS&T bright beads for all channels from all instruments were checked daily, and flow cytometer performances were maintained aligned for the entire study.

#### Panel of reagents/antibodies

CEC were stained by a recently published panel of reagents^7^, that have been listed and detailed in Supplemental Table 3. In order to achieve a high level of standardization, liquid reagents for the panel and the related control tube were lyophilized (Supplemental Table 3) as previously reported^11,12^. A single lyophilized-reagent tube lot was used all along the study (BD Biosciences; cat. 623920).

#### Sample staining

For each sample, 20 × 10^6^ leukocytes were processed as already described^7^ within 4 hours from material collection. In details, the sample volume containing 20 × 10^6^ leukocytes underwent an erythrocyte-lysis step, with 45 mL of Pharm Lyse solution (BD Biosciences) for 15 minutes at RT, under gentle agitation. Samples were then centrifuged (400 g, 10 min, room temperature) and washed by adding 2 mL of Stain Buffer (BD Biosciences). The pellet of each sample was added to the lyophilized cocktail of reagents, previously re-hydrated by the addition of 100 µl of Stain Buffer (BD Biosciences); 1 µM Syto16 (Thermo Fisher Scientific, Eisai, Medipost - US) was finally added, as liquid drop-in, to each tube. Samples were incubated in the dark for 30 minutes at 4 °C, washed with 2 mL of Stain Buffer (BD Biosciences), centrifuged (400 g, 10 min, room temperature), and re-suspended in 1.5 mL of FACSFlow (BD Biosciences).

#### Data acquisition

2–4 × 10^6^ events/sample with lymph-monocyte morphology (as shown in Supplemental Fig. 1) were acquired by flow cytometry (FACSCanto, FACSAria, BD Biosciences) at “medium” flow rate mode, or flow rate = 3 for FACSAria. A threshold combination was used on Forward Scatter (FSC) and Fluorescein isothiocyanate (FITC-Syto16) channels to get rid of very small and non-nucleated events. Compensations were calculated using CompBeads (BD Biosciences) and single stained fluorescent cells. Carryover between samples was prevented by appropriate instrument cleaning at the end of each sample acquisition.

#### Data analysis

CEC were identified as already described^7^; consensus was reached on the final gating strategy, which is shown and detailed in Supplemental Fig. 1; data were centrally collected and analysed by a single operator by using FACSuite v1.04 software (BD Biosciences). To ensure correct gate placement, cells were plotted using dot-plot bi-exponential display^13^.

In order to assess non-specific fluorescence, both fluorescence minus one and isotype controls in combination with all the remaining surface reagents present in the panel were used^7,14,15^.

CEC numbers were calculated by a dual-platform counting method using the lymphocyte subset as reference population (Supplemental Fig. 1Al,m) as previously reported^7,16^.

### Peripheral blood mononuclear cell isolation and cryopreservation

In order to isolate peripheral blood mononuclear cells (PBMC) from freshly drawn PB (n = 3), 8 mL/sample of PB were diluted by adding an equal volume of PBS. Diluted PB was then stratified on 8 mL of Lymphoprep (StemCell Technology, Milano, Italy). Tubes were then centrifuged (1800 g, 20 min, room temperature) and PBMC were harvested and washed. Isolated PBMC were re-suspended in a Foetal Bovine Serum solution containing 10% dimethyl sulfoxide and cryopreserved (−80 °C) for one week^17^. Thawed samples were processed for flow cytometry analysis as above described.

### Statistics

Statistical analyses were performed using XLSTAT ver. 2014.5.03 (Addinsoft, Paris, France), GraphPad Prism ver. 6.07 (GraphPad Software Inc., La Jolla, Ca, USA) and MedCalc ver. 5.00.017 (MedCalc Software, Ostend, Belgium). Parametric or non-parametric tests were used as appropriate. Comparison between males and females was performed using Mann–Whitney *U* test. Paired *t*-test or Sign test were used for paired samples. Statistical comparison among healthy subjects, lSSc and dSSc was performed applying the Kruskal-Wallis test, followed by Dunn’s multiple comparison test. The statistical analysis for biological variability evaluation was carried out as already described^18^. The analytical coefficient of variation (CV_A_), the intra-subject biological coefficient of variation (CV_I_) and the inter-subject biological coefficient of variation (CV_G_) values were calculated. Both index of individuality (II) and critical difference (Cd) values were derived from analytical and biological variability data^19,20^. II yields information about the utility of conventional population-based reference intervals^19,20^, while Cd represents the minimal clinically significant variation when repeated analyses are carried out on the same patient^18,21,22^. In order to evaluate the heterogeneity of the obtained intra-subject variability values, the heterogeneity index (HI) was calculated^23,24^. Statistical significance was accepted at P < 0.05 (two tailed).

## Results

### Flow cytometry standardization of CEC identification and count

In order to demonstrate the standardization of the whole method, reagents, protocols, data acquisition and analysis were standardized. Antibody lyophilisation and the use of same material lots guarantied the standardization of reagents. The alignment of instrument performances was obtained, sustained and monitored by the calculation of Median Fluorescence Intensity (MedFI) values for CS&T bright beads for all channels, from all instruments, all along the study (as detailed in the method section). In addition, as suggested by the EuroFlow working-group^25^, the evaluation of protocol standardization was obtained by monitoring MedFI values of antigens expressed at stable levels on specific subsets of cells. Table 1 shows the Inter-Laboratory CV (CV_L_) of the MedFI calculated for CD34bright expression on CEC (CD34_CEC_) and CD45 on Lymphocytes (CD45_Lympho_), where CD45 allowed the CEC double platform counting. Average of MedFIs was calculated using 6 samples/site, randomly selected at the beginning (the first six months), and 6 samples/site randomly selected at the end (the last six months) of the study. Therefore, CV_L_ values were obtained from MedFI averages, both for CD34_CEC_ (CV_L_Beginning_ = 33.40%, CV_L_End_ = 28.98%) and for CD45_Lympho_ (CV_L_Beginning_ = 20.42%, CV_L_End_ = 22.56%). MedFI of CD146 expression on CEC and relative CV_L_ values were not included in such analysis, since the intensity of the surface expression of this marker on CEC resulted highly spread and characterized by a relevant biological heterogeneity, therefore it did not result useful for the demonstration of the reached standardization level. As shown, the obtained MedFI CV_L_ values, measuring the performance alignment of the different sites of the core network, resulted largely comparable to previously reported data for similar studies, allowing us to ascertain the standardization of the pre-analytical phase^25^. The consistency among sites of the post-analytical phase was reached by the collection and the analysis of all acquired data by a single operator, as suggested^25,26^.

**Table 1:** Inter-laboratory coefficients of variation (CV_L_).

MedFI drift and MedFI variation					
Cell Subset	Marker	Beginning (n = 36)		End (n = 36)	
		MedFI Average	CV_L_%	MedFI Average	CV_L_%
CEC	CD34_CEC_ PE-Cy7	14644.89	33.40	11466.89	28.98
Lymphocytes	CD45_Lympho_ APC-H7	7474.61	20.42	6003.39	22.56

Median Fluorescence Intensity (MedFI) values were obtained for CD34 bright expression on CEC (CD34_CEC_) and for CD45 on lymphocytes (CD45_Lympho_). Averages of MedFI were calculated from 6 samples/site randomly selected at the beginning (the first six months), and 6 samples/site randomly selected at the end (the last six months) of the study. Therefore, inter-laboratory Coefficient of Variation (CV_L_) was calculated both for CD34_CEC_ and CD45_Lympho_, from MedFI averages.

### CEC normality ranges in healthy subjects

**Figure 1:**
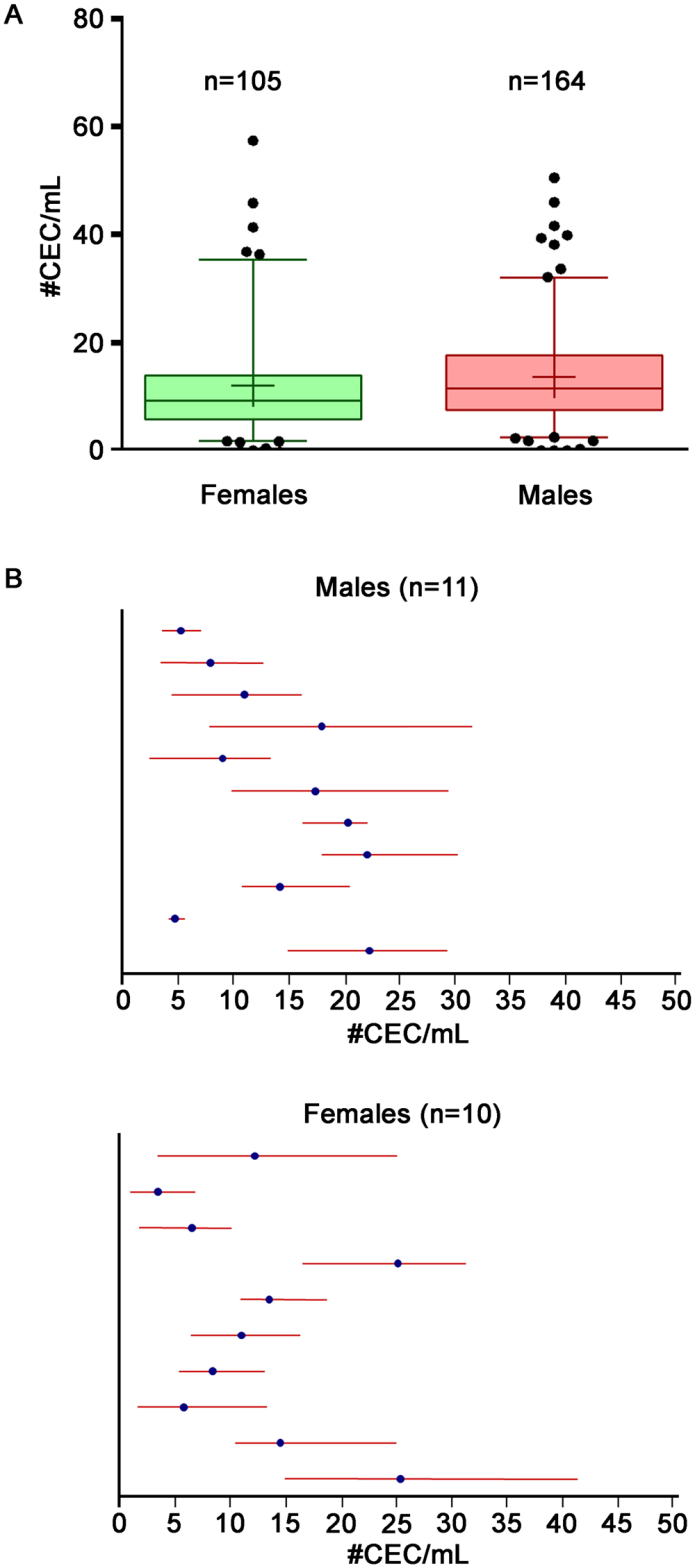
Numbers of CEC and Biological variability in healthy subjects. Panel A. Box plots refer to CEC numbers obtained for female (n = 105) and male (n = 164) donors. Boxes encompass values from the first quartile (bottom) to the third quartile (top). Horizontal line represents median value, cross, mean value. Whiskers represent 5–95^th^ percentile. Mann–Whitney *U* test was used. Panel B. CEC numbers (#CEC/mL) were assessed at four time points (once a week, for four consecutive weeks) on PB samples from 21 healthy subjects enrolled by all the core network sites for the biological variability evaluation. Means and ranges are shown for males (n = 11) or females (n = 10) separately.

In order to establish CEC normality ranges, a population of 269 healthy donors was enrolled across the six sites involved in the network (Supplemental Table 2). CEC absolute count was assessed as above described and numbers are listed in Table 2 and summarized in Fig. 1A. Even if similar, CEC counts resulted significantly different when genders were compared (P = 0.0218). In particular, in the female group (n = 105), CEC numbers ranged between 1.85 and 35.40 cells/mL (5–95^th^ percentile) with a median of 9.31 CEC/mL; in the male population (n = 164), CEC numbers ranged between 2.53 and 32.04 cells/mL (5–95^th^ percentile) with a median of 11.55 CEC/mL (Table 2). Healthy PB samples collected from 53 of the 269 already analysed subjects were also assayed after 3 months. The range of CEC numbers at the first evaluation overlapped with CEC numbers obtained after three months (Supplemental Table 4), suggesting that, in the healthy population, CEC ranges resulted particularly stable overtime. Spearman correlation analysis revealed that age, weight, height and body max indexes (BMI) did not correlate with CEC numbers.

**Table 2:** Absolut numbers of CEC from PB of healthy donors (n = 269).

Statistic	#CEC/mL	
	Females	Males
No. of observations	105	164
Minimum	0.00	0.00
Maximum	57.46	50.60
1st Quartile	5.79	7.55
Median	9.31	11.55
3rd Quartile	13.95	17.67
Mean	12.10	13.69
Standard deviation	10.02	9.19
5^th^ Percentile	1.85	2.53
95^th^ Percentile	35.40	32.04

### Analytical and biological variability of CEC counts

In order to assess the physiological variability of CEC counts, a total of 21 healthy donors were evaluated by the six sites of the network, at four different time points (once a week, for four consecutive weeks). Figure 1B shows averages and absolute intervals of CEC numbers for the 21 analysed donors, then used for the calculation of the intra- and inter-subject biological variability and of the related derived parameters (Table 3). The intra-subject biological coefficient of variation values (CV_I_) reflected relevant biological within-subject variability (CV_I_ for all subjects = 46.3%) in terms of CEC count, resulting slightly higher in the female (51.9%) than in the male group (41.6%).

**Table 3:** Biological variability of CEC count.

Group (n)	CEC/mL (mean)	CV_A_ %	CV_I_ %	CV_G_ %	II	Cd %	HI
All (21)	13.24	14.8	46.3	46.5	1.01	134.6	0.42
Male (11)	13.84	14.6	41.6	45.4	0.86	122.13	0.52
Female (10)	12.58	15.7	51.9	50.2	1.08	150.19	0.36

Mean values, analytical coefficient of variation (CV_A_), intra-subject biological coefficient of variation (CV_I_), inter-subject biological coefficient of variation (CV_G_), index of individuality (II), critical difference (Cd) and heterogeneity index (HI) for CEC counts.

Index of individuality (II) = SD_S_^2^/SD_G_^2^; critical difference (Cd) = [2.77(CV_A_^2^ + CV_I_^2^)^1/2^]; heterogeneity index (HI) = 1 − CV_S_/[(2/k − 1)^1/2^];

SD_S_^2^ = average of within-subject total variance; SD_G_^2^ = inter-subject biological variance;

CV_S_ = intra-subject total coefficient of variation; k = number of specimens.

As reported, the calculation of analytical coefficient of variation (CV_A_) and CV_I_ allows the evaluation of the imprecision of the assay^27^. Formulae for the calculation of reference CV_A_ (imprecision thresholds) have been applied as already reported, and allowed the definition of the assay performance as “*optimum*” (for CV_A_ ≤ 11.6), *“desirable”* (for CV_A_ ≤ 23.3) and “*minimum*” (CV_A_ ≤ 34.7). In the case of the method here presented, CV_A_ values (15.7% for females and 14.6% for males) resulted very close to the respective optimal reference value (≤11.6)^27^.

CV_I_ numbers (41.6% for males and 51.9% for females) resulted highly overlapping with the values obtained for the inter-subject biological coefficient of variation (CV_G_), and CV_G_ values resulted slightly higher in females (CV_G_ = 50.2%) than in males (CV_G_ = 45.4%).

Also, these data allowed to calculate other derived parameters, such as the critical difference (Cd), the heterogeneity index (HI), and the index of individuality (II). In our dataset, Cd values (134.6% for all subjects, 150.19% for females and 122.13% for males) resulted particularly high, despite a very good CV_A_. HI was also calculated in order to explain sample heterogeneity, expressed by the intra-subject total coefficient of variation (CV_s_). Values obtained for all the 21 subjects (HI = 0.42) or separately for males (HI = 0.52) and females (HI = 0.36) indicated that there is no significant heterogeneity among intra-subject variances. The II obtained for all subjects was 1.01 (1.08 for females and 0.86 for males), demonstrating the clinical utility of CEC ranges here calculated for the healthy population.

### Sample stability for CEC count

The stability of PB samples (n = 14), in terms of CEC absolute counts, was measured over time by processing the specimens immediately after the bleeding (T_0_) and 24 h after blood collection (T_24_). CEC counts significantly decreased after 24 h of sample storage (Supplemental Fig. 2A; P = 0.0021, paired t-test).

The stability of CEC numbers after PBMC cryopreservation was also determined. As shown in Supplemental Fig. 2B, the whole CD34bright/CD45neg/CD146pos compartment disappeared after a cryopreservation step, thus making not valuable CEC numbers under these storage conditions.

### Specificity of the method

**Figure 2:**
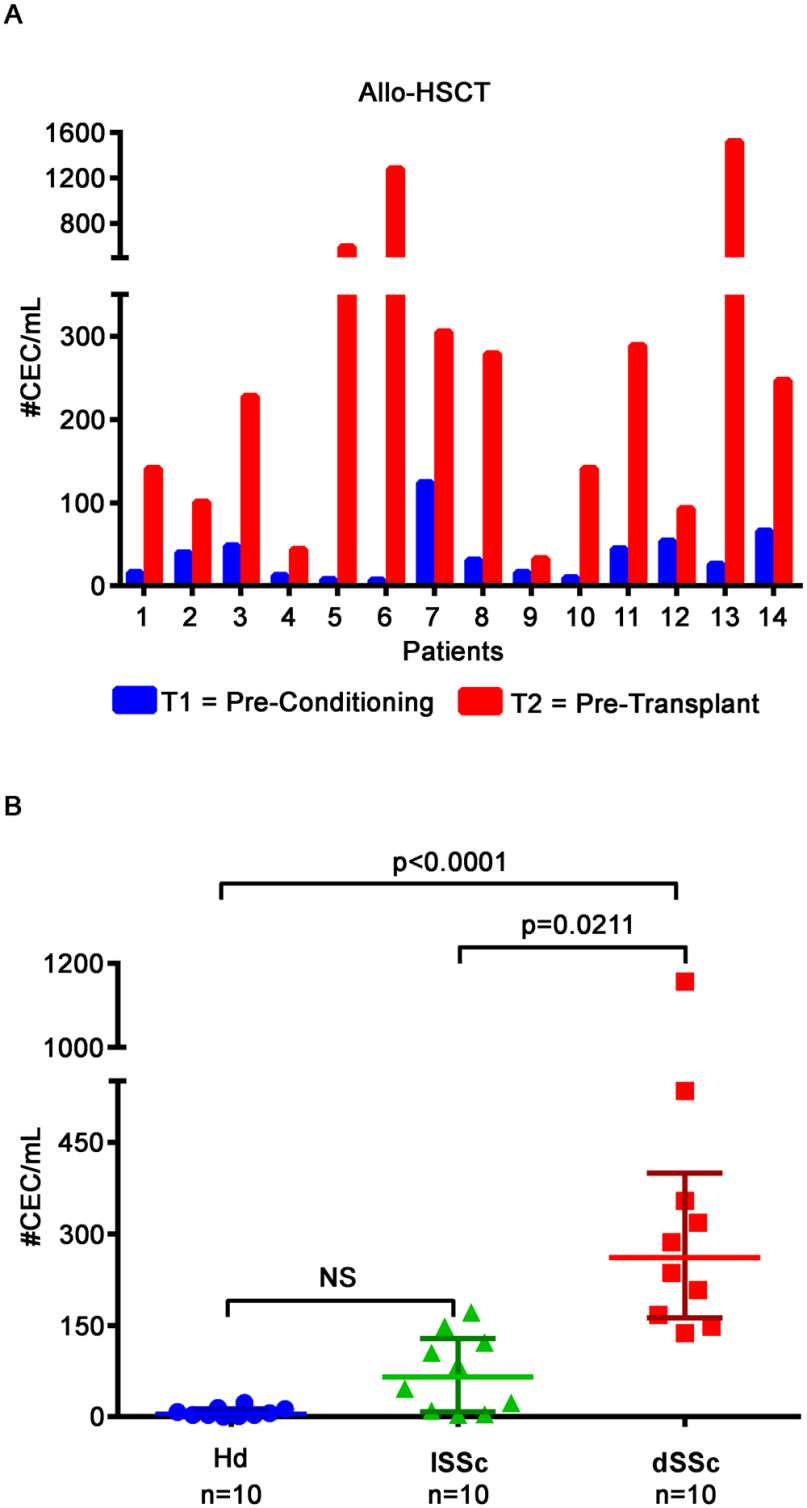
Numbers of CEC in allogeneic-Hematopoietic stem cell transplantation (allo-HSCT) and in cutaneous systemic sclerosis patients. Panel A. Bars display numbers of CEC calculated for allo-HSCT patients (n = 14). Their CEC numbers were evaluated at two different time points: before (T_1_, pre-conditioning, blue bars) and after (T_2_, pre-transplant, red bars) the conditioning regimen. The two aforementioned conditions were compared using the Sign test (P = 0.0001). Panel B. Graphs refer to CEC numbers calculated for healthy donors (Hd, n = 10), limited (lSSc, n = 10) and diffuse (dSSc, n = 10) cutaneous systemic sclerosis patients, enrolled by Site 7. The horizontal median line represents median value; other horizontal lines define the interquartile range. Statistical comparison among healthy subjects, limited and diffuse cutaneous systemic sclerosis patients was performed applying Kruskal-Wallis method (P < 0.0001) followed by Dunn’s multiple comparisons test. NS: not significant.

Two pathological settings in which CEC numbers were expected to be increased have been evaluated as a proof-of-principle that physio-pathological CEC count variations can be monitored, prospectively in clinics, by the method here established. In details, Site 5 of the network, recruited 14 haematological patients undergoing allo-HSCT; these patients were evaluated before (T_1_, pre-conditioning) and at the end of the conditioning regimen (T_2_, pre-transplant) (Fig. 2A). Of note, at T_2_ CEC counts raised to a median value of 237.50 CEC/mL (range = 33.00–1520.00), resulting significantly higher with respect to the CEC numbers in T_1_ (median = 28.50 CEC/mL, range = 7.00–124.00; P = 0.0001, Sign test), accounting for the endothelial damage exerted by the conditioning regimen.

Site 7, originally not included in the core network, assuming fully standardized conditions, recruited 20 systemic sclerosis patients, stratified as limited (lSSc, n = 10) and diffuse (dSSc, n = 10)^10^. In addition, the same site enrolled 10 local internal healthy donors (Hd) that showed CEC number counts (0–23 CEC/mL) falling within the reference range proposed by the network (Fig. 2B). Numbers of CEC, both in lSSc and in dSSc patients resulted higher with respect to Hd. Furthermore, it was observed that in lSSc patients, CEC numbers ranged 3.00–171.00 cells/mL with a median of 65.50 CEC/mL, while in dSSc patients, numbers ranged 137.00–1156.00 cells/mL with a median of 261.00 CEC/mL (Fig. 2B) (Kruskal-Wallis test, P < 0.0001).

## Discussion

A relevant body of literature suggests that the study of CEC has extended the spectrum of cellular biomarkers, since numbers of these cells in the PB might reflect endothelium damage and dysfunction^1,28^. As matter of fact, aberrant CEC counts have been demonstrated in several clinical conditions^2,4,29,30^. Despite these evidences, the translation of basic research into clinical practice has been limited by several issues, especially linked to the application of non-standardized methods that give inconsistent results in terms of CEC definition and count.

Standardization, in the context of PFC assays, allows to decrease variability and subjectivity, that depends on the experience and knowledge of individual operators, as well as on the variable antibody combinations applied in different laboratories^8^. In fact, the main debated problem related to the study of CEC involves their identification, since different panels have been proposed for CEC phenotyping. In this regard, we have recently developed a highly optimized PFC protocol for accurate CEC identification and count^7^, in which these cells were phenotyped as events alive and nucleated, CD34bright/CD45negative/CD146positive. Such antigenic pattern was here confirmed and approved by a network of six different laboratories. Consistency of results underlined that this panel and the related protocol are highly reproducible and therefore suitable for application in network studies^8^. Based on this anchorage point, the aim of the present study was to standardize every single step of the procedure (from the pre-analytical to the data analysis phase) for its application in a multi-site approach, in order to obtain reference ranges for CEC numbers in healthy donors.

Standardization of instruments was obtained, sustained and monitored along the study with bead-based quality controls; this ensured the reproducibility of data measurements across the time, and the stability of the different flow cytometers belonging to the network^8^. By using a lyophilized tube of reagents and same lots of ancillary materials, the possibility of random errors and each single source of analytical verifiable variability were removed. Moreover, since it has been previously reported that the largest single contributor to variability in flow cytometry is the difference in gating establishment by different operators^8,26,31^, we have applied the “mixed model strategy”, based on the concept that locally acquired samples must be finally centrally analysed by a single operator^26^. Under these conditions, our method resulted particularly reliable and reproducible, as demonstrated by the analytical variability coefficient value (CV_A_ = 14.8%).

We also analysed the obtained level of standardization by the evaluation of inter-laboratory CVs (CV_L_), based on MedFI values, calculated for the most stable antigens that allowed us the identification of the principal compartments analysed for CEC identification and count: CD34bright expression on CEC (CD34_CEC_) and CD45 on Lymphocytes (CD45_Lympho_). CV_L_, calculated at the beginning and at the end of the study, resulted overlapping to the ones reported for similar flow cytometry network studies^25^.

The demonstrated level of standardization, allowed us to aggregate data from all sites involved in the network and the analysis of CEC counts in a large cohort of healthy subjects (N = 269) revealed that CEC numbers are gender-related (median_female_ = 9.31 CEC/mL, range 5–95^th^ percentile = 1.85–35.40; median_male_ = 11.55 CEC/mL, range 5–95^th^ percentile = 2.53–32.04), even if this difference could not be of clinical relevance. Of note, 53 of these healthy donors were re-evaluated after 3 months and their CEC numbers remained in the calculated normality ranges suggesting that, in the healthy population, CEC counts resulted highly stable overtime.

Since there are no available studies on CEC biological variability, all the six sites of the core network also assessed both the intra-subject and the inter-subject variability of CEC numbers, critical concepts for the introduction of their detection in clinical practice. Results demonstrated that CEC numbers presented high biological intra-subject and inter-subject variability, as expressed by CV_I_ and CV_G_ values respectively, which resulted both slightly higher for females. Nevertheless, since the CV_A_ demonstrated a particularly high reproducibility of the method, and being CV_I_ and CV_G_ characterized by overlapping values, CEC number normality ranges here calculated for the healthy population resulted absolutely robust and useful for further clinical applications, as demonstrated by the values of Cd, II and HI parameters^18^. Under these conditions, CEC analyses must be carried out on freshly drawn PB samples and, as we also established, cryopreservation is not allowed (the whole CEC compartment disappeared in thaw samples).

Finally, in order to demonstrate the method specificity, we identified two pathological conditions, allo-HSCT and cutaneous systemic sclerosis, in which an impairment of CEC numbers was expected^10,32^. Anyway, it must be noted that in both these pathologies, CEC numbers have been already evaluated, but the use of not standardized methods for CEC detection gave inconsistent results^33–37^. By applying the here described standardized methods, CEC counts resulted significantly higher in allo-HSCT patients after they received the conditioning regimen (T_2_, pre-transplant), in comparison to the analysis of CEC from the same patients at the baseline (T_1_, pre-conditioning). We also observed an increase in terms of CEC numbers when cutaneous systemic sclerosis patients were compared to healthy volunteers. At the same time, in the case of systemic sclerosis patients, the sensitivity of the method here described allowed us to identify, for the first time, a statistically significant difference between lSSc and dSSc patients. Altogether, these data are very useful in demonstrating the high specificity of the described protocol, even if the number of analysed patients does not allow any clinical conclusion.

In summary, we defined, for the first time, a standardized method for CEC identification and count. By analysing a large population of donors, an unprecedented level of data robustness was obtained, and CEC reference numbers for the healthy population were established. Therefore, if from one side these procedures represent a model for any rare cell subset analysis by PFC, from the other side they could be useful as starting point for CEC monitoring in any pathology involving endothelial dysfunctions, both in research and in clinical settings.

## Electronic supplementary material

Supplementary Info
