# Supplementary material for: A standardized flow cytometry network study for the assessment of circulating endothelial cell physiological ranges
Source: Sci Rep. 2018 Apr 11;8:5823. doi: 10.1038/s41598-018-24234-0 (PMC5895616; doi:10.1038/s41598-018-24234-0)
Supplement: Supplementary file 1 — Supplementary Info [file 41598_2018_24234_MOESM1_ESM.docx]

**A standardized flow cytometry network study for the assessment of circulating endothelial cell physiological ranges.**

Paola Lanuti^1,2 #^, Pasquale Simeone^1,2 #^, Gianluca Rotta^3 #^, Camillo Almici^4^, Giuseppe Avvisati^5^, Rosa Azzaro^6^, Giuseppina Bologna^1,2^, Alfredo Budillon^7^, Melania Di Cerbo^5^, Elena Di Gennaro^7^, Maria Luisa Di Martino^8^, Annamaria Diodato^6^, Paolo Doretto^9^, Eva Ercolino^1,2^, Alessandra Falda^9^, Chiara Gregorj^5^, Alessandra Leone^7^, Francesca Losa^8^, Natalia Malara^10^, Mirella Marini^11^, Pasquale Mastroroberto^12^, Vincenzo Mollace^10^, Michele Morelli^13^, Emma Muggianu^8^, Giuseppe Musolino^12^, Arabella Neva^4^, Laura Pierdomenico^1,2^, Silvia Pinna^8^, Giovanna Piovani^14^, Maria Serena Roca^7^, Domenico Russo^15^, Lorenza Scotti^16^, Maria Cristina Tirindelli^5^, Valentina Trunzo^10^, Roberta Venturella^13^, Carlo Vitagliano^7^, Fulvio Zullo^13^, Marco Marchisio^1,2,*^ and Sebastiano Miscia^1,2^.

^1^Department of Medicine and Aging Sciences, University “G.d’Annunzio” Chieti-Pescara, Italy; ^2^Centre on Aging Sciences and Translational Medicine (Ce.S.I.-Me.T.), University “G.d’Annunzio” Chieti-Pescara, Italy; ^3^BD Biosciences Italia, 20090 Milano, Italy; ^4^Laboratory for Stem Cells Manipulation and Cryopreservation, Department of Transfusion Medicine, Spedali Civili of Brescia, Brescia, Italy; ^5^Hematology, Stem Cell Transplantation, Transfusion Medicine and Cellular Therapy, Department of Medicine, Campus Bio-Medico University Hospital, Rome, Italy; ^6^Transfusion Service, Department of Hematology-Oncology and Stem Cell Transplantation Unit, ^7^Experimental Pharmacology Unit, Department of Research, Istituto Nazionale Tumori- IRCCS G. Pascale, Naples, Italy; ^8^Unit of Internal Medicine, Allergy and Clinical Immunology, Department of Medical Sciences “M. Aresu”, University of Cagliari Monserrato, Cagliari, Italy; ^9^Clinical Pathology Laboratory, Department of Laboratory Medicine, AAS5, Pordenone Hospital, Pordenone, Italy; ^10^Department of Health Science University "Magna Graecia" of Catanzaro, Catanzaro, Italy; ^11^Laboratory for Stem Cells Manipulation and Cryopreservation, Department of Transfusion Medicine, Spedali Civili of Brescia, Brescia, Italy; ^12^Department of Experimental and Clinical Medicine, University "Magna Graecia" of Catanzaro, Catanzaro, Italy; ^13^Department of Obstetrics and Gynecology, University "Magna Graecia" of Catanzaro, Catanzaro, Italy; ^14^Department Molecular Medicine and Translational, University of Brescia, Brescia, Italy; ^15^Unit of Blood Diseases and Stem Cell Transplantation, University of Brescia, Brescia, Italy; ^16^Department of Statistics and Quantitative Methods, University of Milano-Bicocca.

# P.L., P.S., and G.R. contributed equally to this study.

| Supplemental Table 1. Key steps of the standardization experiments. | |
| --- | --- |
|  | |
| *Study Design Phase* |  |
| Literature screening for a correct selection of the markers |  |
| Panel Design with proper association of fluorochromes/markers | Supplemental Table 3 |
| 8 Point Titration of reagents |  |
| Definition of appropriate staining controls (fluorescence minus two and fluorescence minus one control, as well as unstained and single stained samples) | Material and Methods |
| Reagent Lyophilization |  |
| Definition of enrolment criteria | Material and Methods |
|  |  |
| *Startup Phase* |  |
| Definition of the standardized setup of the instrument | Material and Methods |
| Lyophilized reagent test |  |
| Training of operators |  |
|  |  |
| *Preanalitical Phase* |  |
| Bleeding standardization (same needles, same collection tubes) | Material and Methods |
| Sample processing within 4hrs from bleeding |  |
| Instrument quality control verification before each acquisition, using the same lot of quality control beads | Material and Methods |
|  |  |
| *Analitical Phase* |  |
| Buffer standardization using the same lot of lysing and staining solutions |  |
| Protocol standardization using a checklist to ensure that every operator perform the same steps in the same manner |  |
| Staining standardization using a single batch of reagents and lyophilized antibodies |  |
| Instrument standardization using the same instrument setting, the same acquisition template and acquisition criteria |  |
| Staining of 20 x 10^6^ leukocytes |  |
| Acquisition of 2-4 x 10^6^ lympho-mono cells |  |
|  |  |
| *Data Analysis Phase* |  |
| Consensus on gating strategy | Supplemental Figure 1 |
| Analysis done by a single operator |  |
| Use of biexponential display |  |
| Use of internal positive and negative control | HSC population is a useful negative control for CD146 staining. Activated Lymphocytes are a convenient positive control for CD146 staining. |
| Use of fluorescence minus two controls | Material and Methods |
| Report of analysis in terms of absolute cell counting (double platform in our case) |  |
| Evaluation of the reached standardization level |  |
| Analysis of the analytical and biological variability in terms of CEC count |  |

| Supplemental Table 2. Demographic characteristics of healthy donors (n = 269). | | | | | | | | |
| --- | --- | --- | --- | --- | --- | --- | --- | --- |
| Statistic | **Age** | | **Weight (Kg)** | | **Height (cm)** | | **BMI** | |
|  | **Females** | **Males** | **Females** | **Males** | **Females** | **Males** | **Females** | **Males** |
| No. of observations | 105 | 164 | 105 | 164 | 105 | 164 | 105 | 164 |
| Minimum | 18.00 | 19.00 | 40.00 | 55.00 | 147.00 | 162.00 | 16.02 | 18.99 |
| Maximum | 64.00 | 64.00 | 100.00 | 135.00 | 178.00 | 194.00 | 37.64 | 42.61 |
| 1st Quartile | 28.00 | 30.25 | 55.50 | 72.00 | 160.00 | 172.00 | 20.55 | 23.66 |
| Median | 38.00 | 40.00 | 60.00 | 80.00 | 165.00 | 178.00 | 22.49 | 25.33 |
| 3rd Quartile | 47.50 | 49.00 | 67.00 | 88.75 | 168.50 | 181.00 | 24.98 | 27.76 |
| Mean | 38.13 | 40.09 | 62.53 | 81.27 | 163.94 | 177.09 | 23.26 | 25.89 |
| Standard deviation | 11.27 | 10.89 | 10.33 | 12.91 | 6.35 | 6.26 | 3.61 | 3.72 |
| 5^th^ Percentile | 22.30 | 24.00 | 49.30 | 65.00 | 152.30 | 168.00 | 19.27 | 21.15 |
| 95^th^ Percentile | 58.10 | 58.00 | 84.10 | 108.75 | 174.70 | 188.75 | 30.37 | 32.54 |

| **Supplemental Table 3.** **List of flow cytometry specificities and reagents.** | | | | | |
| --- | --- | --- | --- | --- | --- |
| Detection | Fluorochrome/  Reagent | Vendor | Ab Clone | Catalogue Number | Amount per Test |
| DNA | Syto16 | Thermo Fisher Scientific | - | S-7578 | 1 µM |
| CD146 | PE | BD Biosciences | P1H12 | 623920* | 6 ng |
| Viability | 7-AAD | BD Biosciences | - | 623920* | 12 ng |
| CD34 | PE-Cy7 | BD Biosciences | 8G12 | 623920* | 50 ng |
| CD45 | APC-H7 | BD Biosciences | 2D1 | 623920* | 100 ng |
| VEGFR2 | AlexaFluor647 | BD Biosciences | 89106 | 623920* | 50 ng |
| * Catalogue number of the lyophilized combination.  Keys: R-phycoerythrin (PE); 7-AminoActinomycin D (7-AAD), PE-Cyanine 7 (Cy7), Allophycocyanin-Hilite®7 (APC-H7). Becton Dickinson (BD) Biosciences (San Jose, CA, USA); Life technologies (Monza, Italy). | | | | | |

| **Supplemental Table 4. Comparison of CEC numbers at T_0_ and after 3 months.** | | | | |
| --- | --- | --- | --- | --- |
| Statistic | T_0_ | | After 3 months | |
|  | Males (N = 23) | Females (N= 30) | Males (N = 23) | Females (N= 30) |
| Mean | 12.84 | 9.04 | 13.34 | 12.47 |
| Median | 9.66 | 7.97 | 11.69 | 10.53 |
| 5^th^ Percentile | 2.57 | 0.41 | 3.59 | 0.81 |
| 25^th^ Percentile | 7.20 | 5.00 | 7.79 | 7.24 |
| 75^th^ Percentile | 17.84 | 11.08 | 17.03 | 14.03 |
| 95^th^ Percentile | 32.14 | 25.85 | 29.00 | 33.89 |
| Numbers refer to numbers of CEC/mL of peripheral blood. | | | | |

**SUPPLEMENTAL FIGURES**

**
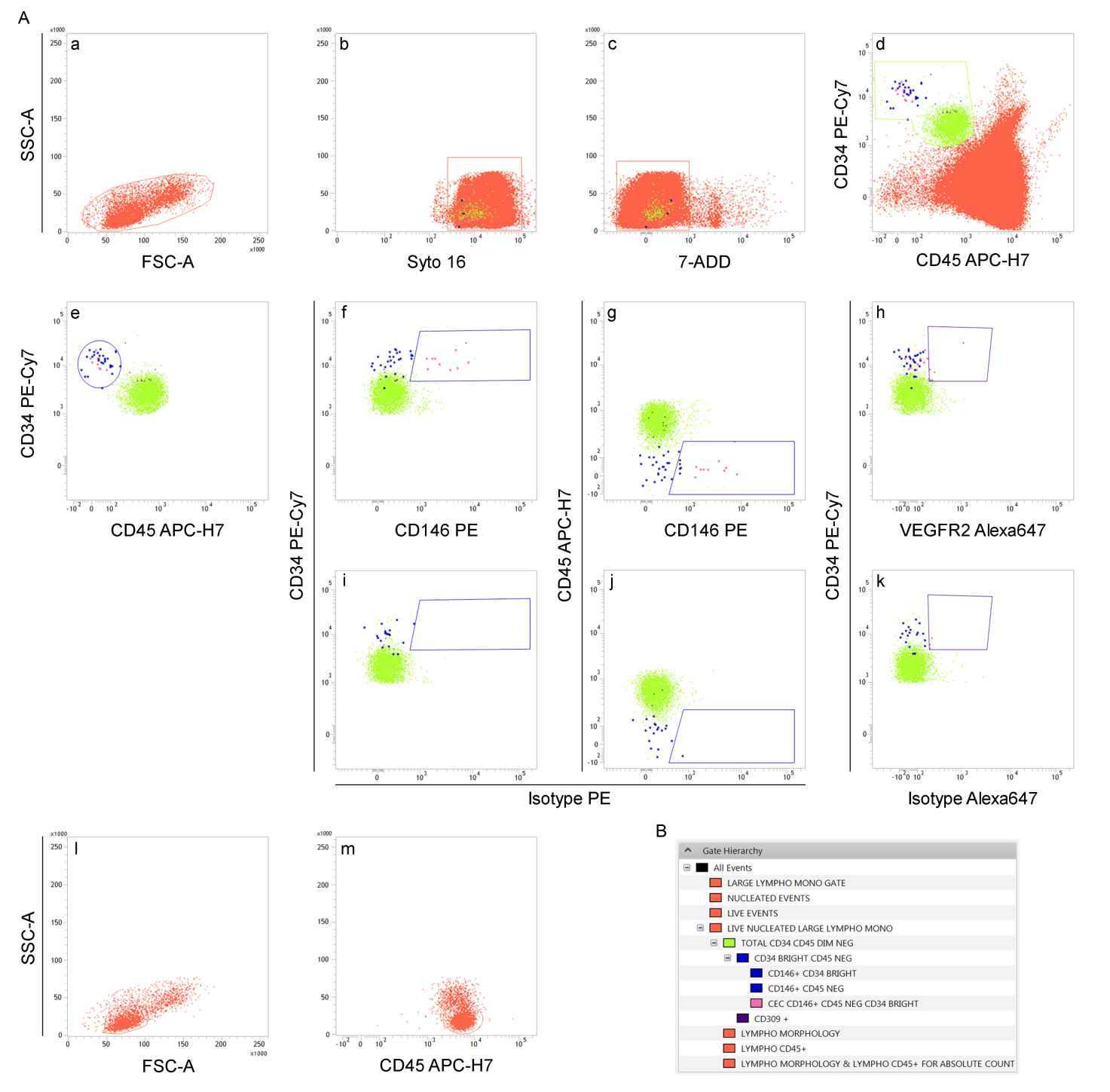
**

**Supplemental Figure 1. Gating Strategy. Panel A. (a)** Lympho-monocytes were gated on a FSC-A/SSC-A dot plot. **(b)** Nucleated events (Syto16pos) were selected (Syto16/SSC-A dot plot) and **(c)** dead cells were excluded (7-AAD/SSC-A dot plot). **(d)** Events characterized by lympho-monocytes features, alive, nucleated, expressing bright levels of CD34 and CD45neg were identified. CD34pos/CD45dim-neg events were observed **(e)** and analysed for CD146 surface expression both on a CD146/CD34 **(f)** and on a CD146/CD45 **(g)** dot plot. The CEC compartment (CD34bright/CD45neg/CD146pos cells, highlighted pink dots) was identified; **(h)** HSC and CD34bright/CD45neg cell populations were analysed for VEGFR2 surface expression, on a VEGFR2/CD34 dot plot. Gates in **f**, **g**, **h** were drawn based on the signal of the control tube, containing the corresponding isotype control in combination with all the remaining surface reagents (**i**, **j**, **k**, respectively). In order to obtain CEC and HSC absolute numbers by double platform counting method, lymphocytes were identified both on the basis of their morphology (**l**, FSC-A/SSC-A dot plot) and on the bright expression of CD45 (**m**, CD45/SSC-A dot plot). Images are representative of 269 healthy PB samples. **Panel B.** The hierarchy of the gating strategy is shown (n = 269).

**
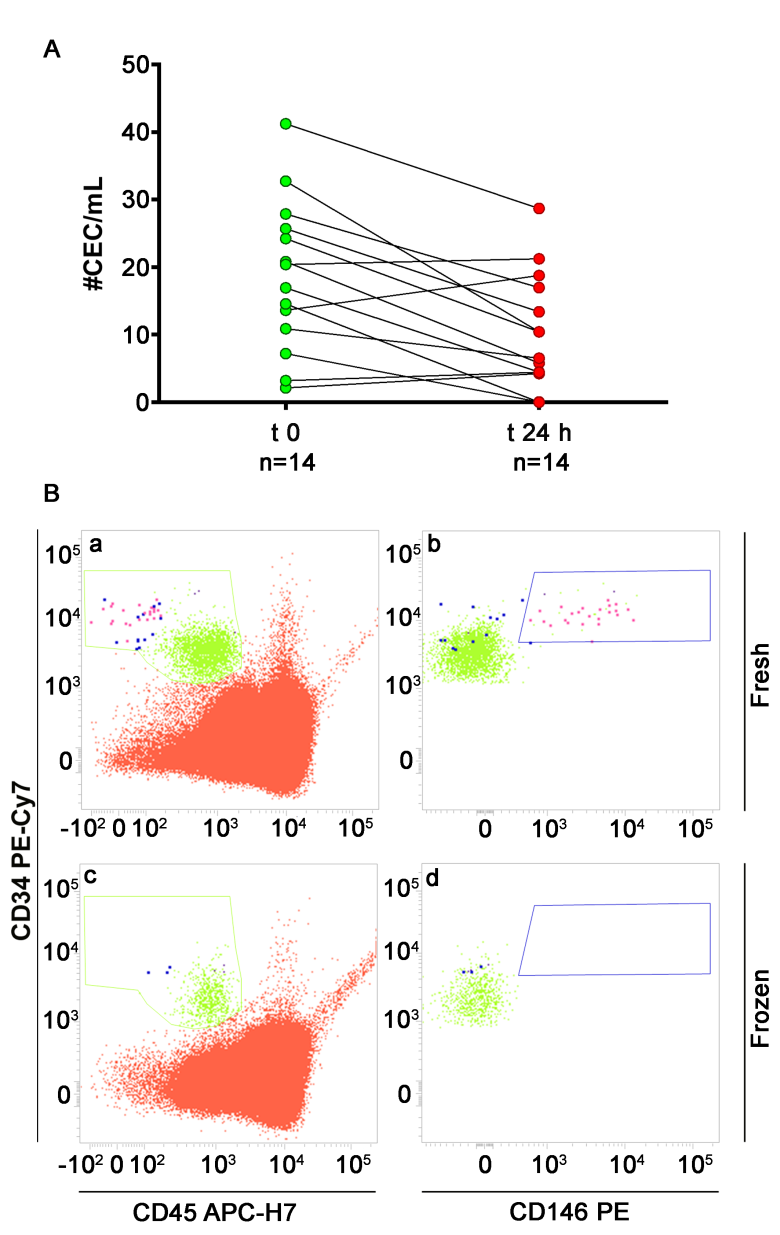
**

**Supplemental Figure 2. Sample storage. Panel A.** Dots represent numbers of CEC obtained from PB of 14 healthy donors analysed immediately after the bleeding (T_0_) or at 24h of storage (T_24_). Each line connects T_0_ and T_24_ of the same donor. The two aforementioned conditions were compared by using the paired t-test (P = 0.0021). **Panel B.** PBMC from three healthy donors were stained immediately after gradient separation (Fresh) and after a cryopreservation step (Frozen). Events displaying the typical lymph-monocyte morphology, alive and nucleated were selected as above described and plotted on a CD45/CD34 dot plot. (**a**) CD34pos/CD45dim-neg events from fresh samples were gated (green gate), analysed for their CD146 expression on a CD146/CD34 dot plot (**b**) and compared to the same dot plots obtained for the respective frozen samples (**c**) and (**d**). Images are representative of three separate experiments.
